# An Alternate Method of Classifying Allergic Bronchopulmonary Aspergillosis Based on High-Attenuation Mucus

**DOI:** 10.1371/journal.pone.0015346

**Published:** 2010-12-15

**Authors:** Ritesh Agarwal, Ajmal Khan, Dheeraj Gupta, Ashutosh N. Aggarwal, Akshay K. Saxena, Arunaloke Chakrabarti

**Affiliations:** 1 Department of Pulmonary Medicine, Postgraduate Institute of Medical Education and Research, Chandigarh, India; 2 Department of Radiodiagnosis, Postgraduate Institute of Medical Education and Research, Chandigarh, India; 3 Department of Medical Microbiology, Postgraduate Institute of Medical Education and Research, Chandigarh, India; Abramson Research Center, United States of America

## Abstract

**Background and Aim:**

Allergic bronchopulmonary aspergillosis (ABPA) is classified radiologically based on the findings of central bronchiectasis (CB) and other radiologic features (ORF). However, the long-term clinical significance of these classifications remains unknown. We hypothesized that the immunological activity and outcomes of ABPA could be predicted on HRCT chest finding of high-attenuation mucus (HAM), a marker of inflammatory activity. In this study, we evaluate the severity and clinical outcomes of ABPA with different radiological classifications.

**Methods:**

Patients were classified based on CT chest findings as: (a) serologic ABPA (ABPA-S) and ABPA-CB; (b) ABPA-S, ABPA-CB, and ABPA-CB-ORF; and, (c) ABPA-S, ABPA-CB and ABPA-CB-HAM. The clinical, spirometric and serological (total and *A fumigatus* specific IgE levels, eosinophil count) severity of the disease and clinical outcomes in various classifications were analyzed.

**Results:**

Of the 234 (123 males, 111 females; mean age, 34.1 years) patients, 55 (23.5%) had normal HRCT, 179 (76.5%) had CB, 49 (20.9%) had HAM, and 27 (11.5%) had ORF. All immunological markers were consistently higher in the HAM classification, while in other classifications these findings were inconsistent. On multivariate analysis, the factors predicting frequent relapses were presence of HAM (OR 7.38; 95% CI, 3.21–17.0) and CB (OR 3.93; 95% CI, 1.63–9.48) after adjusting for ORF.

**Conclusions:**

The classification scheme based on HAM most consistently predicts immunological severity in ABPA. Central bronchiectasis and HAM are independent predictors of recurrent relapses in ABPA. Hence, HAM should be employed in the radiological classification of ABPA.

## Introduction

Allergic bronchopulmonary aspergillosis (ABPA) is a complex immunological disorder that commonly complicates the course of patients with asthma and cystic fibrosis. The disease occurs secondary to antigens released by *Aspergillus fumigatus*, an ubiquitous fungi that colonizes the tracheobronchial tree in these patients.[1], [2] The condition clinically presents with poorly controlled asthma, hemoptysis, weight loss and fever. The Rosenberg-Patterson criteria are most often used for diagnosis.[3], [4] High-resolution computed tomography (HRCT) of thorax is the imaging modality of choice for the diagnosis of ABPA. The findings on HRCT chest include central bronchiectasis (CB), high-attenuation mucus (HAM), air trapping (mosaic attenuation) and centrilobular nodules. The prevalence of ABPA in asthma is as high as 19% whereas the prevalence in cystic fibrosis ranges from 6–10%.[2], [5] The first case of ABPA was reported in 1952 by Hinson from the United Kingdom (UK)[6] whereas in the United States (US) it was identified in 1967.[7] There was an initial belief that the disorder is rare in North America[8] but subsequent reports disproved this myth.[9], [10] In fact, amongst the four largest series of ABPA in the world (111 cases [UK][11]; 118 cases [US][4]; 155 cases [India][12], 164 [India][13]), one is from the United States.

There are patients of ABPA who otherwise fulfill all diagnostic criteria, but lack demonstrable abnormalities on CT chest. They are labeled as seropositive ABPA (ABPA-S) compared with the more common presentation with CB (ABPA-CB).[14] Another CT classification scheme categorizes ABPA into mild (ABPA-S), moderate (ABPA-CB) and severe (ABPA-CB with other radiologic findings [ABPA-CB-ORF]).[15] The active inflammatory component of ABPA clinically manifests as excess mucus secretion and radiologically as mucoid impaction.[16] On HRCT images, the CT attenuation of mucoid impaction in ABPA is similar to or less than the attenuation of paraspinal skeletal muscle (CT Hounsfield values, 10–40).[17], [18], [19] However, in many patients, mucoid impaction manifests with high attenuation CT values (CT Hounsfield values, 70–170),[12], [18], [20] and is visually denser than the paraspinal skeletal muscle.[12], [18], [20], [21], [22], [23], [24] The criterion for HAM is based on the visual appearance of the density of mucus being greater than the paraspinal skeletal muscle. The corresponding high CT attenuation values wherever available further strengthens the diagnosis. The clinical importance of HAM lies in the fact that it has been shown to be associated with recurrent relapses.[12], [25]

Over the last two decades there is an increased awareness that CT findings can predict outcomes in many pulmonary disorders including CF.[26], [27], [28], [29] We hypothesized that the presence of HAM on CT chest at diagnosis would not only correlate with immunological severity, but could also predict outcome in ABPA. Herein, we assess the severity of the disease and clinical significance of different radiological classifications based on HRCT chest findings, and also propose a new radiologic classification for ABPA.

## Materials and Methods

The present study is a post hoc analysis of prospectively collected data, and includes 234 consecutive patients of ABPA diagnosed between January 2004 and December 2008 and followed till December 2009. The clinical characteristics of 205 patients have been previously described. [12], [25], [30] The study was approved by the Institute Ethics Committee, PGIMER, Chandigarh, and a written informed consent was taken from all patients.

In our Chest Clinic, all patients with asthma (except those with glucocorticoid intake more than three weeks in the preceding six months) are screened for *Aspergillus* sensitization using an intradermal skin test. Patients who demonstrate immediate cutaneous hyperreactivity to *Aspergillus* antigen are further investigated for ABPA with IgE levels (total and *A fumigatus* specific), eosinophil count, *Aspergillus* precipitins and HRCT chest. The detailed methodology has been previously described.[12], [25], [30] Patients are diagnosed as ABPA if they meet both the following criteria: (a) total IgE levels >1000 IU/mL; (b) *A fumigatus* specific IgE levels >0.35 kUA/L; and, two of the following criteria: (a) presence of serum precipitins against *A fumigatus*; (b) radiographic pulmonary opacities (fixed/transient); (c) absolute eosinophil count >1000 cells/µL; (d) central bronchiectasis on HRCT.[12], [25], [30], [31]

The HRCT of the chest was categorized for the presence and extent of bronchiectasis.[32] Bronchiectasis was classified as ‘central’ when confined to the medial half (point midway between hilum and chest wall) of the lung.[33] The presence of HAM was considered if the mucus was visually denser than the normal paraspinal skeletal muscle and the corresponding CT attenuation values were noted.[12], [24] Other radiologic findings (ORF) such as pulmonary fibrosis, bleb, bullae, pneumothorax, parenchymal scarring, emphysematous change, multiple cyst, fibrocavitary lesions and pleural thickening proposed by Kumar et al. were recorded.[15] Based on the HRCT findings the patients were classified according to the following schemes:

(a) Greenberger classification: ABPA-S and ABPA-CB[14]

(b) Kumar classification: ABPA-S, ABPA-CB and ABPA-CB-ORF[15]

(c) Classification based on HAM: ABPA-S, ABPA-CB and ABPA-CB-HAM

The patients were treated with glucocorticoids and followed up with history and physical examination, chest radiograph and total IgE levels every six weeks.[2] Treatment response was classified as *remission*, if the IgE levels declined by >35% and there was clinical/radiological improvement after three months of glucocorticoids or *relapse* if there was a doubling of baseline IgE levels with clinicoradiological worsening. Patients with two or more relapses were classified as *frequent relapses* whereas those with no or one relapse were categorized as *infrequent relapses*. For the purpose of this study, we evaluated the clinical and serological severity in the three radiological classifications, and the factors predicting frequent relapses. To ascertain the clinical significance of various classifications, we systematically excluded patients with HAM, CB and ORF from each of the classifications and then re-analyzed the severity.

### Statistical analysis

Data are presented as mean (SD), median (IQR) or number (percentage). Statistical significance was assumed at a p-value of less than 0.05. The differences between continuous variables were analyzed using student's t-test, Mann-Whitney U test, ANOVA (with Scheffe's test for post hoc analysis) or Kruskal-Waalis test (with Dwass-Steel-Critchlow-Fligner test for post hoc analysis) as appropriate. Categorical variables were compared using the chi-square test. A multivariate logistic regression analysis was performed to determine the CT findings predicting frequent relapses.

## Results

**Figure 1:**
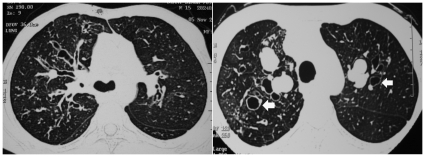
Presence of central bronchiectasis in two different patients with allergic bronchopulmonary aspergillosis. The presence of classic signet ring appearance of dilated bronchi is easily appreciable (arrows). The bronchiectasis is located predominantly in the inner half of the lung fields.

**Figure 2:**
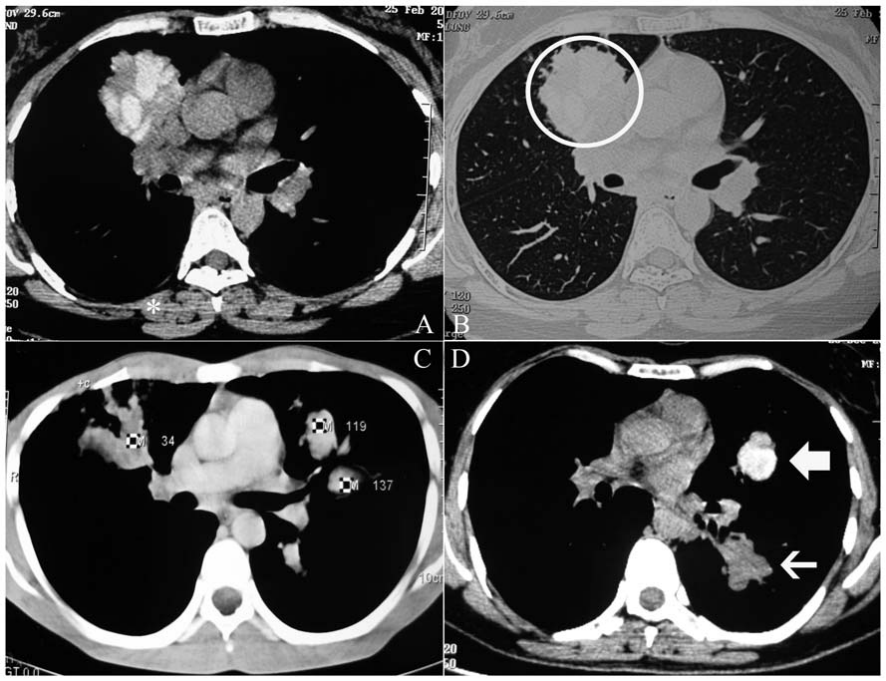
High-resolution computed tomographic images of patients with allergic bronchopulmonary aspergillosis demonstrating the presence of high-attenuation mucus. (A) Mediastinal window showing the presence of hyperattenuated mucus within dilated bronchi. The mucus is denser than the paraspinal skeletal muscle (asterisk) (B) Lung window shows that hyperdense mucus can occasionally be appreciated even with the parenchymal sections (circle); (C) CT Hounsfield values of mucus in dilated bronchi: mucus in the left lung is hyperdense with higher CT attenuation values compared to mucoid impaction in the right lung; (d) Hyperattenuated (bold arrow) and normal attenuation mucus (thin arrow) in the same mediastinal window.

**Figure 3:**
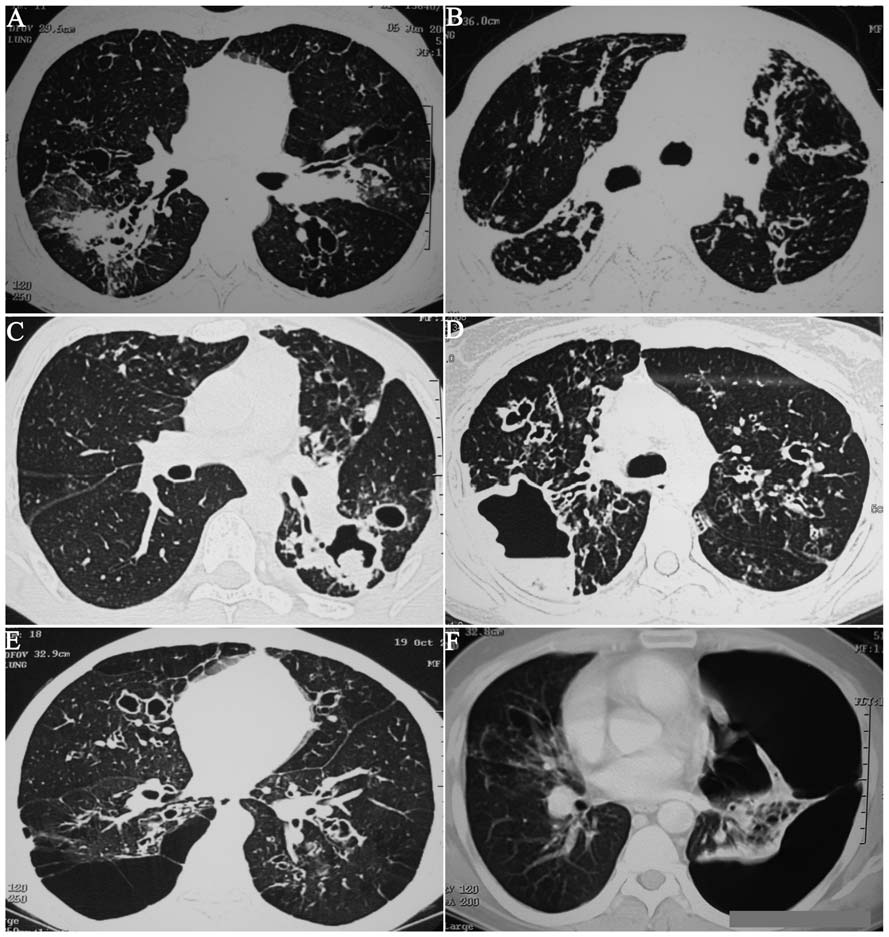
Other radiological features (ORF) that are encountered in patients with allergic bronchopulmonary aspergillosis: (A) parenchymal fibrosis involving the right lower lobe; (B) parenchymal and pleural fibrosis; (C) aspergilloma within a bronchiectatic cavity; (D) large cavity with air fluid level; (E) multiple bulla with central bronchiectasis; (F) left sided pneumothorax- numerous bronchiectatic cavities can be appreciated in the collapsed lung.

There were 123 males and 111 females with a mean (SD) age of 34.1 (12.5) years. The baseline characteristics of these patients are shown in Table 1. The median duration of asthma was six years with the recognition of ABPA being generally delayed. In fact, 40.6% of patients had inappropriately received antitubercular therapy in the past. Fifty-five (23.5%) patients had a normal HRCT (ABPA-S) and 179 (76.5%) had CB (ABPA-CB) (Figure 1). Forty-nine (20.9%) patients were identified to have HAM impaction on HRCT chest (Figure 2) at presentation whereas 27 (11.5%) patients had ORF (Figure 3). Three patients had HAM involving a single lobe whereas the remaining had multilobar involvement. The density measurements are available in only 15 patients and ranged from 108 to 168 Hounsfield units.

**Table 1:** Baseline characteristics including spirometry, serological and HRCT findings (n = 234).

**Demographic details**	
Age (years), mean (SD)	34.1 (12.5)
Male Gender, No. (%)	123 (52.6)
**History**	
Duration of Asthma (years), median (IQR)	6 (4–13)
Hemoptysis, No. (%)	86 (36.8)
Expectoration of brownish black mucous plugs, No. (%)	93 (39.7)
Cigarette smoking, No. (%)	14 (6)
History of anti-tuberculous therapy, No. (%)	95 (40.6)
**Spirometry, No. (%)**	
Normal	55 (23.5)
Mild obstruction	59 (25.2)
Moderate obstruction	75 (32.1)
Severe obstruction	45 (19.2)
Bronchodilator reversibility	103 (44)
**Serological findings**	
*Aspergillus skin test, No. (%)*	
Type I	234 (100)
Type III	181 (77.4)
Absolute eosinophil count (cells/µL), median (IQR)	847 (480–1551)
*Aspergillus* precipitins, No. (%)	194 (82.9)
Total IgE levels (IU/mL), median (IQR)	5015 (2839–10000)
*A fumigatus* specific IgE levels (kUA/L), median (IQR)	4.6 (1.4–16.1)
**HRCT findings, No. (%)**	
Normal	55 (23.5)
*Central bronchiectasis*	179 (76.5)
No. of lobes, median (IQR)	3 (2–4)
No. of segments, median (IQR)	7 (5–10)
Other radiologic findings (pulmonary fibrosis, bleb, bullae, pneumothorax, parenchymal scarring, emphysematous change, multiple cyst, fibrocavitary lesions and pleural thickening)	27 (11.5)
Centrilobular nodules and/or tree-in-bud opacities	69 (29.5)
High attenuation mucus	49 (20.9)

### Greenberger classification

The clinical features, spirometry and serological findings as per the Greenberger classification is shown in Table 2. The *A fumigatus* specific IgE levels and eosinophil counts but not the total IgE levels were higher in ABPA-CB compared to ABPA-S. However, the serological severity varied once patients with HAM and ORF were excluded from the analysis. ***On removal of ORF***, both *A fumigatus* specific IgE levels and eosinophil counts remained significant, while ***on removal of HAM*** only the eosinophil counts retained significance (Table 2). The clinical history and the spirometry findings were similar in the two groups. The numbers of relapses were higher in the CB group irrespective of the presence or absence of HAM and ORF.

**Table 2:** Clinical, spirometric, serological differences and outcomes in patients with allergic bronchopulmonary aspergillosis (ABPA) based on the classification by Greenberger et al with and without the presence of high attenuation mucus (HAM) and other radiologic findings (ORF).

		All patients (n = 234)		Without HAM (n = 185)		Without ORF (n = 207)	
Characteristics	ABPA-S (n = 55)	ABPA-CB (n = 179)	P value	ABPA-CB (n = 130)	P value	ABPA-CB (n = 152)	P value
***Clinical history***							
Age, in years	35.9 (13.4)	33.6 (12.2)	0.23	33.9 (12.3)	0.37	34.1 (11.9)	0.36
Male Gender, No.(%)	25 (45.5)	98 (54.7)	0.23	73 (56.2)	0.18	83 (54.6)	0.24
Duration of asthma, in years	6 (5–12)	6 (4–14)	0.85	7 (4–15)	0.75	7 (4–15)	0.82
***Spirometry*** *,* No.(%)							
Normal	15 (27.3)	40 (22.3)	0.33	21 (16.2)	0.05	36 (23.9)	0.55
Mild obstruction	14 (25.5)	45 (25.1)		34 (26.2)		39 (25.7)	
Moderate obstruction	20 (36.4)	55 (30.7)		39 (30)		48 (31.6)	
Severe obstruction	6 (10.9)	39 (21.8)		36 (27.7)		29 (19.1)	
***Serological findings***							
Total IgE levels, IU/mL	3850 (2650–7800)	5400 (2859–10313)	0.07	4689 (2619–7895)	0.65	5457 (2867–10314)	0.06
*A fumigatus* specific IgE levels, kUA/L	2.1 (0.95–9.5)	4.8 (1.7–18)	0.02	3.4 (1.4–13.8)	0.19	5.6 (1.9–17.9)	0.01
Absolute eosinophil count, cells/µL	620 (250–962)	983 (558–1770)	0.001	850 (475–1506)	0.03	1048 (650–1800)	0.0001
Type III AST, No.(%)	45 (81.8)	136 (76)	0.37	98 (75.4)	0.34	120 (79.5)	0.2
*Aspergillus* precipitins, No.(%)	48 (87.3)	146 (82)	0.36	103 (79.8)	0.23	117 (77)	0.46
***Clinical outcome***							
Number of relapses	0 (0–1)	2 (0–2)	0.0001	1 (0–2)	0.004	2 (0–2)	0.0001
Frequent (≥2) relapses, No. (%)	8 (14.5)	89 (49.7)	0.0001	49 (37.6)	0.002	75 (49.3)	0.0001

Values are expressed as mean (SD) or median (IQR) unless otherwise stated; ABPA-S - serologic ABPA; ABPA-CB - ABPA with central bronchiectasis.

The *A fumigatus* specific IgE levels and eosinophil counts were higher in ABPA-CB compared to ABPA-S. The serological severity varies once patients with HAM and ORF are excluded. On removal of HAM (column 4), only the eosinophil counts retain significance, whereas on removal of ORF (column 6), both *A fumigatus* specific IgE levels and eosinophil counts remain significant.

### Kumar classification

On classifying the patients according to the Kumar classification (Table 3), the clinical history and spirometric findings were similar in the three groups. The total IgE levels were statistically insignificant between the groups. The *A fumigatus* specific IgE levels and eosinophil counts were significantly different between the groups. On post hoc analysis, the *A fumigatus* specific IgE levels were higher in ABPA-CB vs. ABPA-S and the eosinophil counts were higher in ABPA-CB compared to the other two groups (ABPA-S and ABPA-CB-ORF). ***Once patients with HAM were excluded***, only the eosinophil counts retained significance in patients with ABPA-CB compared to ABPA-S and ABPA-CB-ORF. The number of relapses were higher in both ABPA-CB and ABPA-CB-ORF compared to ABPA-S, but was not different between ABPA-CB and ABPA-CB-ORF.

**Table 3:** Clinical, spirometric, serological differences and outcomes in patients with allergic bronchopulmonary aspergillosis (ABPA) based on the classification by Kumar et al with and without the presence high attenuation mucus (HAM).

		All patients (n = 234)			Without HAM (n = 185)		
Characteristics	ABPA-S (n = 55)	ABPA-CB (n = 152)	ABPA-CB-ORF (n = 27)	P value	ABPA-CB (n = 111)	ABPA-CB-ORF (n = 19)	P value
***Clinical history***							
Age, in years	35.9 (13.4)	34.1 (11.9)	30.4 (13.7)	0.16	34.2 (11.9)	32 (14.9)	0.49
Male Gender, No.(%)	25 (45.5)	83 (54.6)	15 (55.6)	0.48	61 (55)	12 (63.2)	0.33
Duration of asthma, in years	6 (5–12)	7 (4–15)	5 (3–10)	0.18	8 (4–15)	5 (3–8)	0.08
***Spirometry*** *,* No.(%)							
Normal	15 (27.3)	36 (23.7)	4 (14.8)	0.21	20 (18)	3 (15.8)	0.25
Mild obstruction	14 (25.5)	39 (25.7)	6 (22.2)		30 (27)	4 (21.1)	
Moderate obstruction	20 (36.4)	48 (31.6)	7 (25.9)		34 (30.6)	5 (26.3)	
Severe obstruction	6 (10.9)	29 (19.1)	10 (37)		27(24.3)	7 (36.8)	
***Serological findings***							
Total IgE levels, IU/mL	3850 (2650–7800)	5457 (2867–10314)	5400 (2320–10220)	0.18	4588 (2500–7860)	5800 (2659–11000)	0.36
*A fumigatus* specific IgE levels, kUA/L	2.1 (0.95–9.5)	5.6 (1.9–17.9)^c^	2.3 (1.4–18.3)	0.03	3.9 (1.4–13)	1.6 (1.1–18.3)	0.35
Absolute eosinophil count, cells/µL	620 (250–962)	1048 (650–1800)^c^^,^ d	450 (200–985)	0.0001	983 (593–1540)^c^^,^ d	370 (161–650)	0.0001
Type III AST, No.(%)	45 (81.8)	117 (77)	19 (70.4)	0.5	84 (75.7)	14 (73.7)	0.62
*Aspergillus* precipitins, No.(%)	48 (87.3)	120 (79.5)	26 (96.3)	0.06	85 (77.3)	18 (94.7)	0.09
***Clinical outcome***							
Number of relapses	0 (0–1)	2 (0–2)^c^	2 (0–2)^a^	0.0001	1 (0–2)^c^	1 (0–2)	0.03
Frequent (≥2) relapses, No. (%)	8 (14.5)	75 (49.3)^c^	14 (51.8)^a^	0.0001	42 (37.8)^c^	7 (36.8)^a^	0.007

Values are expressed as mean (SD) or median (IQR) unless otherwise stated; ABPA-S - serologic ABPA; ABPA-CB - ABPA with central bronchiectasis; ABPA-CB-ORF - ABPA with CB and other radiologic findings;

a ABPA-CB-ORF value significant compared to ABPA-s;

b ABPA-CB-ORF value significant compared to ABPA-CB;

c ABPA-CB value significant compared to ABPA-s;

d ABPA-CB value significant compared to ABPA-CB-ORF;

The *A fumigatus* specific IgE levels and eosinophil counts, but not the total IgE levels are different between the groups (columns 1 to 3). On post hoc analysis, the *A fumigatus* specific IgE levels are higher in ABPA-CB (column 2) compared to ABPA-S (column 1) and the eosinophil counts are higher in ABPA-CB (column 2) in comparison to the other two groups (columns 1 and 3). Once HAM is excluded (columns 5 and 6), only the eosinophil counts remain significant in ABPA-CB (column 5) compared to ABPA-S (column 1) and ABPA-CB-ORF (column 6).

### New classification based on HAM

On classifying patients based on HAM (Table 4), the eosinophil counts and the IgE levels (total and *A fumigatus* specific) were higher in ABPA-CB-HAM compared to both ABPA-S and ABPA-CB, and remained significant even after exclusion of patients with ORF (Table 5). The clinical history and the spirometric findings were similar in the three groups. The numbers of relapses were not only higher in both ABPA-CB and ABPA-CB-HAM compared to ABPA-S but were also higher in ABPA-CB-HAM vs. ABPA-CB.

**Table 4:** Clinical, spirometric, serological differences and outcomes in patients with allergic bronchopulmonary aspergillosis (ABPA) based on the proposed staging on the basis of high attenuation mucus (HAM) with and without the presence of other radiologic findings (ORF).

		All patients (n = 234)			Without ORF (n = 207)		
Characteristics	ABPA-S (n = 55)	ABPA-CB (n = 130)	ABPA-CB-HAM (n = 49)	P value	ABPA-CB (n = 107)	ABPA-CB- HAM (n = 45)	P value
***Clinical history***							
Age, in years	35.9 (13.4)	33.9 (12.3)	32.6 (12)	0.33	34.6 (11.9)	33 (11.9)	0.37
Male Gender, No.(%)	25 (45.5)	73 (56.2)	25 (51)	0.4	59 (55.1)	24 (53.3)	0.5
Duration of asthma, in years	6 (5–12)	7 (4–15)	5 (3–11)	0.28	7 (4–15)	5 (3.5–12)	0.27
***Spirometry*** *,* No.(%)							
Normal	15 (27.3)	21 (16.2)	10 (20.4)	0.25	19 (17.8)	10 (22.2)	0.48
Mild obstruction	14 (25.5)	34 (26.2)	11 (22.4)		29 (27.6)	10 (22.2)	
Moderate obstruction	20 (36.4)	39 (30)	16 (32.7)		33 (30.8)	15 (33.3)	
Severe obstruction	6 (10.9)	36 (27.7)	12 (24.5)		26 (24.3)	10 (22.2)	
***Serological findings***							
Total IgE levels, IU/mL	3850 (2650–7800)	4689 (2619–7895)	10220 (5310–12200)^a^^, ^ b	0.0001	4590 (2698–7860)	10314 (5556–12435) a ^, ^ b	0.0001
*A fumigatus* specific IgE levels, kUA/L	2.1 (0.95–9.5)	3.4 (1.4–13.8)	7.8 (4.7–23.7)^a^^, ^ b	0.0001	3.9 (1.4–13)	9.3 (4.7–23.9) a ^, ^ b	0.0001
Absolute eosinophil count, cells/µL	620 (250–962)	850 (475–1506)	1200 (800–2506)^a^^, ^ b	0.0001	983 (593–1540)	1200 (824–2056) a ^, ^ b	0.0001
Type III AST, No.(%)	45 (81.8)	98 (75.4)	38 (77.6)	0.63	82 (76.6)	35 (77.8)	0.75
*Aspergillus* precipitins, No.(%)	48 (87.3)	103 (79.8)	43 (87.8)	0.3	81 (76.4)	39 (86.7)	0.15
***Clinical outcome***							
Number of relapses	0 (0–1)	1 (0–2)^c^	3 (2–3)^a^^, ^ b	0.0001	1 (0–2)^c^	3 (2–3)^a^^, ^ b	0.0001
Frequent (≥2) relapses, No. (%)	8 (14.5)	49 (37.7)^c^	40 (81.6)^a^^, ^ b	0.0001	39 (36.4)^c^	36 (80)^a^^, ^ b	0.0001

Values are expressed as mean (SD) or median (IQR) unless otherwise stated; ABPA-S - serologic ABPA; ABPA-CB - ABPA with central bronchiectasis;

a ABPA-CB-HAM value significant compared to ABPA-s;

b ABPA-CB-HAM value significant compared to ABPA-CB;

c ABPA-CB value significant compared to ABPA-s;

d ABPA-CB value significant compared to ABPA-HAM.

**Table 5:** Effects of HRCT findings (HAM, number of bronchiectatic segments and ORF) on occurrence of frequent relapses - multivariate logistic regression model.

Variables	Adjusted odds ratio (95% confidence intervals)	P value
High-attenuation mucus	6.86 (3.03 to 15.52)	**0.0001**
Other radiologic findings	1.34 (0.56 to 3.21)	0.505
Central bronchiectasis	3.41 (1.45 to 8.01)	**0.005**
***Constant***	1.043	0.901

### Impact of serological and CT findings on frequent relapses

There was clinical and radiological improvement in all the patients receiving glucocorticoid therapy. The mean (SD) duration of follow-up was 31.1 (17.6) months. 138 (59%) patients experienced relapse of ABPA with the median (range) relapses being 1 (0–7). There were 97 (41.5%) patients with frequent relapses. On multivariate logistic regression analysis, the factors predicting frequent relapses were presence of HAM and CB after adjusting for other CT finding of ORF (Table 5).

## Discussion

The results of this study suggest that HRCT chest findings correlate with immunological severity in ABPA, and the most consistent marker of serological severity was HAM. The classification of ABPA based on other criteria was inconsistent with some features being severe in one classification but not in the other. The CT findings of HAM and CB at diagnosis were also independent predictors of recurrent relapses.

The Rosenberg-Patterson criteria (Table 6) are most frequently used in the diagnosis of ABPA.[3], [4] However, there is lack of consensus on the number of criteria required for diagnosis or the specific cut-off values for the various tests utilized in diagnosis.[34] An immediate cutaneous hypersensitivity to *A fumigatus* antigen is the most reliable method for screening for ABPA.[5], [31], [35], [36], [37] A positive test is virtually seen in all patients although 40% of asthmatics without ABPA can also demonstrate skin test positivity.[5] However, the type III and IV cutaneous reactions do not have diagnostic or prognostic value.[2], [12], [25], [30] The total IgE level is another useful test in the diagnosis of ABPA, and normal values exclude active ABPA from the work-up of patients with respiratory symptoms. Neither the initial levels nor the quantum decline in IgE values have any prognostic significance.[12], [25] Elevated *A fumigatus* specific IgG/IgE levels greater than twice the pooled serum samples from patients with *Aspergillus* hypersensitive asthma is paramount in the differential diagnosis of ABPA from *Aspergillus* sensitized asthma.[30], [38] Serum precipitins to *A fumigatus* although present in 69–90% of patients with ABPA,[11], [12], [39], [40], [41] are also seen in 10% of other pulmonary disorders including asthma,[41], [42], [43] and thus represent supportive not diagnostic evidence for ABPA.

**Table 6:** Rosenberg-Patterson criteria for the diagnosis of allergic bronchopulmonary aspergillosis [3], [4].

*Major criteria*
• Bronchial asthma
• Immediate cutaneous hypersensitivity to *A fumigatus* antigen
• Serum total IgE levels (>1000 IU/mL)
• Serum *A fumigatus* specific IgG and/or IgE levels more than twice the mean plus two standard deviation values in patients with Aspergillus hypersensitive asthma
• Central bronchiectasis on HRCT chest
• Serum precipitins against *A fumigatus*
• Fleeting or fixed pulmonary opacities on chest radiograph
• Peripheral blood eosinophil count >1000 cells/µL
*Minor criteria*
• Sputum cultures demonstrating growth of *A fumigatus*
• Expectoration of brownish-black mucus plugs
• Type III skin reactions to *A fumigatus* antigen

The presence of six out eight major criteria makes the diagnosis almost certain.

Similarly, there is no agreement on the severity classification, and they continue to be modified and updated.[31], [34] Greenberger et al. classified ABPA into ABPA-S or ABPA-CB respectively depending on the absence or presence of bronchiectasis.[14] They proposed that patients with ABPA-S represent the earliest stage of the disease with less severe immunologic findings compared to ABPA-CB.[14] However, in this study only the *A fumigatus* specific IgG levels and precipitins were higher in patients with ABPA-CB.[14] The other immunologic parameters namely total IgE and *A fumigatus* specific IgE were similar in the two groups.[14] Thereafter Kumar et al. divided ABPA into three categories and suggested that ABPA-CB-ORF represents clinically and serologically the severest form of ABPA.[15] However, this study had included only 18 patients (six in each group). Moreover, the findings included in the categorization of ABPA-CB-ORF were all changes representing the fibrotic stage of the disease. Both the studies were limited by the sample size and the fact that the clinical significance of these classifications was not investigated.

In this study, we evaluated the clinical and serological severity of both these classifications using data from a large set of patients. The classification scheme by Greenberger et al. showed immunological severity in some parameters (eosinophil count and *A fumigatus* specific IgE levels) but not in others (total IgE levels). Moreover, on excluding patients with HAM, the immunological severity was restricted only to eosinophil counts. Interestingly, in the Kumar classification, the immunological markers were most severe in patients with ABPA-CB and not ABPA-CB-ORF. This suggests that ORF does not determine serological severity and probably represents the fibrotic, burnt out phase of the disease. In addition to evaluating these two classifications, we proposed a new classification based on HAM.[16], [44], [45] The HAM classification was the most consistent with immunological severity persisting even after removal of patients with ORF.

High attenuation mucous plugging is described as the most characteristic finding of ABPA.[22], [46], [47] The finding is attributed either to the presence of calcium salts, iron and manganese,[48] or desiccated mucus.[49] The association of HAM with poorer outcomes in ABPA remains unclear, but one obvious reason is that the higher attenuation points to a more inspissated type of mucus. It is probable that the presence of HAM defines a subgroup of patients with more severe inflammation. Numerous genetic alterations have been described with ABPA,[2] and it may well be hypothesized that patients with HAM have certain genetic abnormalities that dictate a disease with more severe inflammation and poorer outcomes. However, more research is needed to investigate the exact reason for this association. There was no specific anatomical/geometric occurrence and distribution of HAM observed in this study. In contrast to previous series that found HAM to be more prevalent in unilobar mucus plugging,[18] most of our patients had multilobar hyperdense mucus plugging.

We also assessed the clinical significance of all classifications in terms of relapse. The presence of CB was a consistent marker of relapse in all classifications irrespective of whether patients with HAM or ORF were included or not. Moreover, in the Kumar classification, there was no statistical significance in the relapse rates between ABPA-CB and ABPA-CB-ORF, suggesting that it is the CB and not ORF that predicts relapse. However, in the HAM classification, the relapse rates were higher in ABPA-CB-HAM compared to ABPA-CB even after excluding patients with ORF suggesting that HAM independently predicts relapses. Finally, this was also confirmed in the multivariate model, wherein CB and HAM were independent predictors of frequent relapses.

Few studies have described factors predicting outcome in patients with ABPA. In two earlier studies, we had observed that HAM and CB are independent markers of relapse.[12], [25] Greenberger et al. and Kumar et al. reported that patients with ABPA-S developed fewer exacerbations and no patient progressed to irreversible lung disease.[14], [15], [50] However, the studies by Greenberger and Kumar did not include all the CT findings. Also, these studies did not perform multivariate analysis to determine factors predicting frequent relapses, which was performed in our study.[14], [15], [50]

The current study is different from our previous reports in numerous aspects, although it does contain majority of patients from these previous studies.[12], [25], [30] The outcome used for defining relapses is different from our previous reports.[12], [30] Previously, we had defined relapse as doubling of the baseline IgE levels irrespective of the patient's symptoms or appearance of radiological opacities.[12], [25], [30] However, we have observed that doubling of IgE levels could also occur non-specifically and hence in this study we have used a definition that includes doubling of IgE levels as well as clinicoradiological worsening. Another important difference is the definition of ORF used in this study. In our previous study, we had defined ORF as that originally described by Kumar et al. plus other CT features such as centrilobular nodules, tree-in-bud opacities and HAM.[30] However, in this study the definition of ORF is strictly retained as defined by Kumar et al.[15] This strict compartmentalization has been performed to clearly ascertain the significance of ORF vis-à-vis HAM in defining the radiological severity and their significance in ABPA. Finally the multivariate regression model in this study includes only CT findings and not serological findings, because we had previously demonstrated that serological findings do not predict clinical outcome.[12], [25]

What are the clinical implications of this study? Based on the results of this study, a patient with ABPA and hyperattenuated mucus at diagnosis represents an immunologically severe disease with increased propensity of recurrent relapses. Whether these group of patients require more intensive treatment protocols or closer monitoring remains to be answered. Hence, HAM should be incorporated in the classification of ABPA. These conclusions are valid and particularly relevant, not only because of the large sample size and long duration of follow-up, but also due to the fact that we analyzed the clinical, serological and long-term outcomes in terms of relapses.

In conclusion, the classification scheme based on high attenuation mucus most consistently predicts immunological severity in ABPA. CB and HAM are independent predictors of frequent relapses in ABPA. Hence, HAM should be employed in the radiological classification of ABPA.
